# Supplementary material for: Highly cross-linked carbon tube aerogels with enhanced elasticity and fatigue resistance
Source: Nat Commun. 2023 Jun 1;14:3178. doi: 10.1038/s41467-023-38664-6 (PMC10235059; doi:10.1038/s41467-023-38664-6)
Supplement: Supplementary file 1 — Supplementary Information [file 41467_2023_38664_MOESM1_ESM.pdf]

Supplementary Materials

Highly cross-linked carbon tube aerogels with enhanced elasticity and fatigue resistance

*Lei Zhuang[1], De Lu[1], Jijun Zhang[1], Pengfei Guo[1], Lei Su[1], Yuanbin Qin[1], Peng Zhang[1], Liang Xu[1], Min Niu[1], Kang Peng[1], Hongjie Wang[1]**

[1] State Key Laboratory for Mechanical Behavior of Materials Xi'an Jiaotong University, Xi'an, 710049, China

*E-mail: hjwang@xjtu.edu.cn;

Supplementary Materials include:
Supplementary Fig. 1 to 12
Supplementary Table 1
Supplementary References

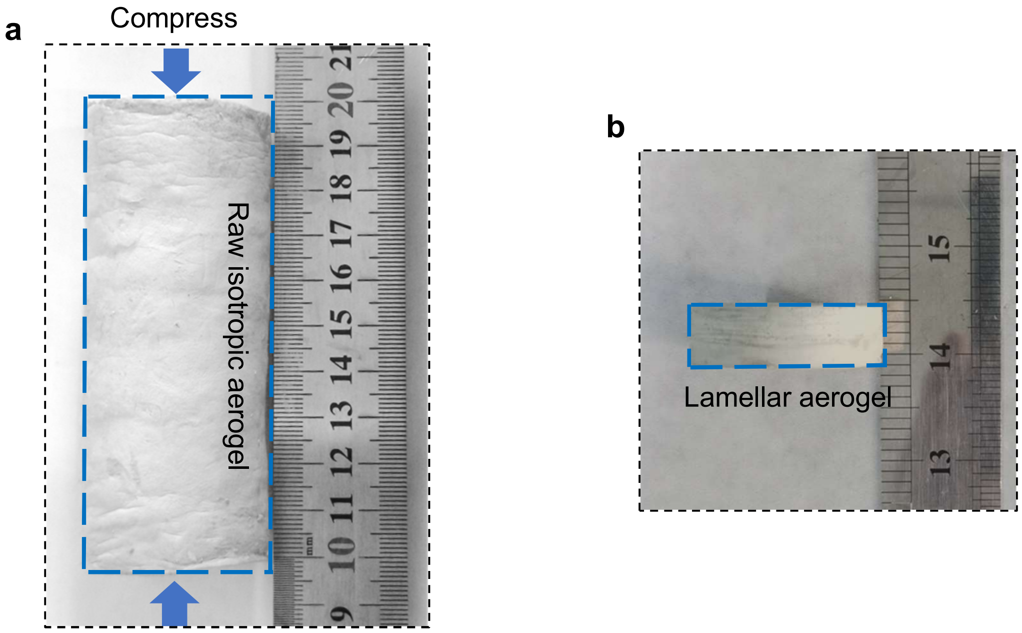

Supplementary Fig. 1. Preparation of the lamellar SiC nanowire aerogels. (a) A raw SiC nanowire aerogel with a density of ~10 mg cm^{-3}. (b) A lamellar SiC nanowire aerogel with a density of ~200 mg cm^{-3}) after being hot-pressed at 1100 °C for 2 h with a pressure of ~10 MPa.

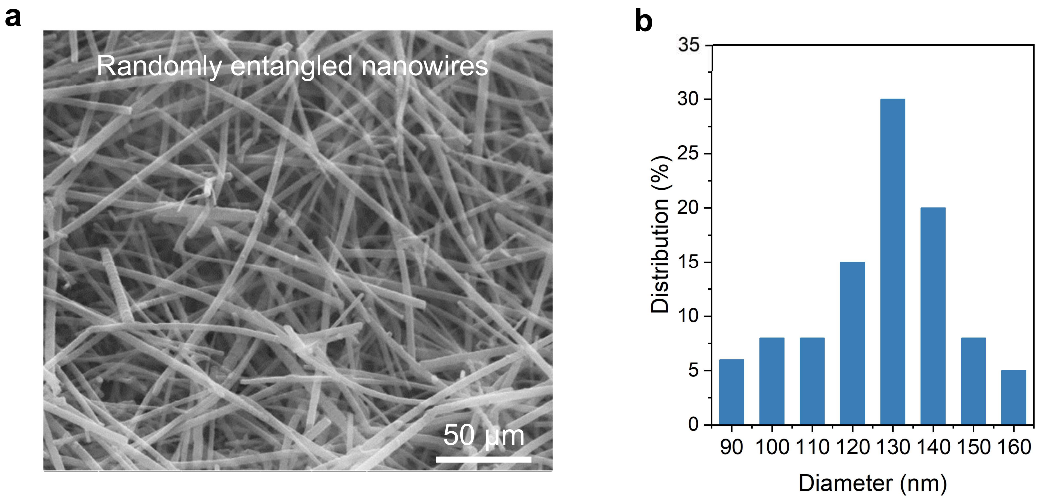

Supplementary Fig. 2. Microstructure of the raw SiC nanowire aerogels with a density of ~10 mg cm^{-3}. (a) SEM image of the sample surface, in which nanowires are distributed randomly. (b) Diameter distribution of SiC nanowires in a range of 90–160 nm.

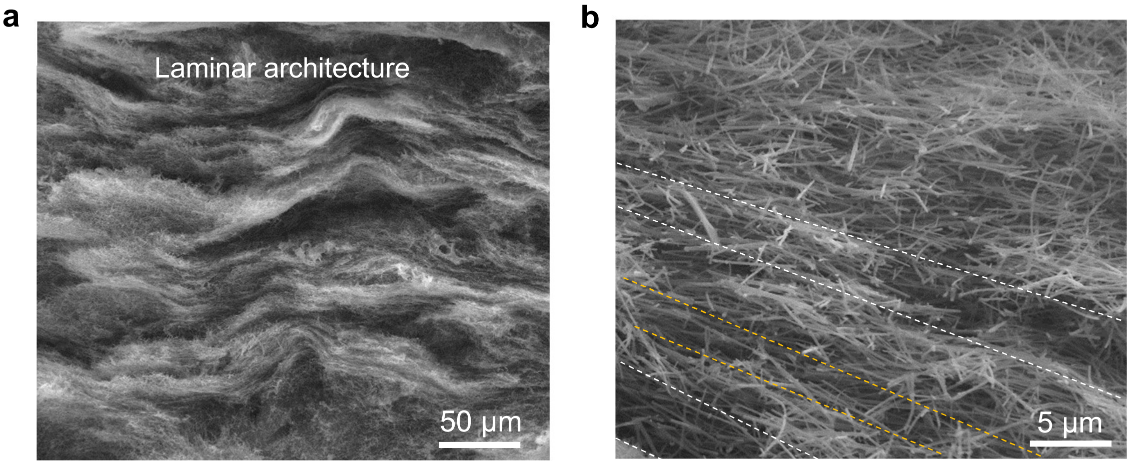

Supplementary Fig. 3. Microstructure of the lamellar SiC nanowire aerogels after hot-pressing with a density of ~200 mg cm^{-3}. (a) Low resolution SEM image. (b) High resolution SEM image.

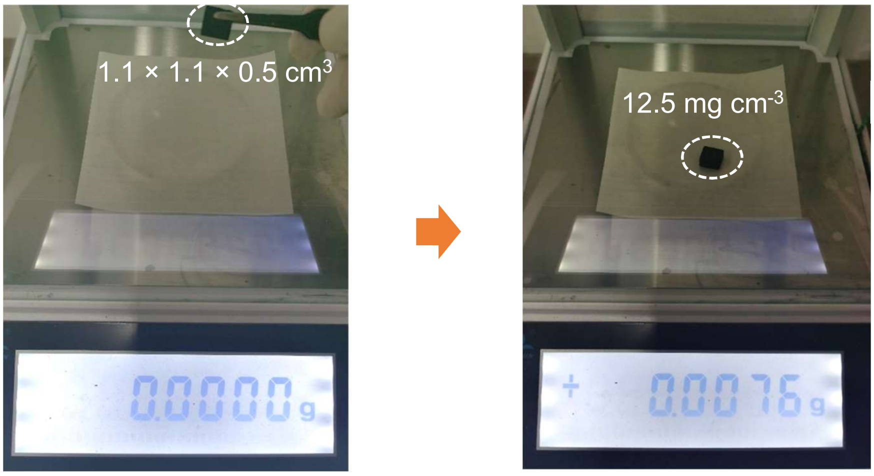

Supplementary Fig. 4. Density measurement of a CTA. A lightweight CTA with a density measured to be 12.5 mg cm^{-3}.

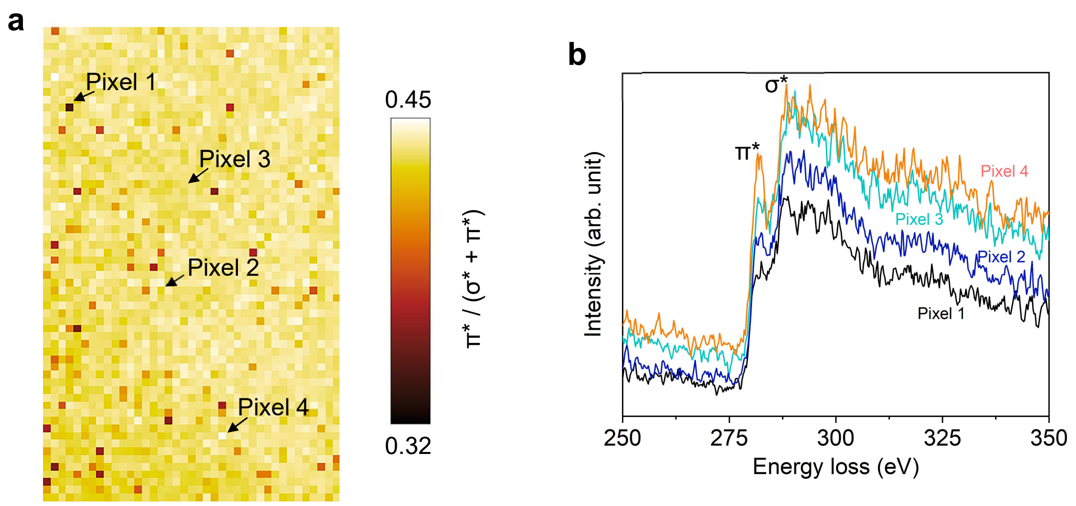

Supplementary Fig. 5. Typical EELS spectra obtained from a single carbon tube. (a) EELS mapping with pixels that shows the ratio of π* to (π* + σ*) using two-window intensity-ratio method[1]. (b) Raw curve data drew from the selected pixels, which show different peak intensity of π* and σ*. According to atomic orbital theory, each threefold (or sp^2) site contributes one state to the π* band and three states to the σ* band; while each fourfold (or sp^3) site contributes a total four states to the σ* band. The different peak intensity ratio of π* to σ* bands may indicate the variety of sp^2 and sp^3 bonds along the tube.

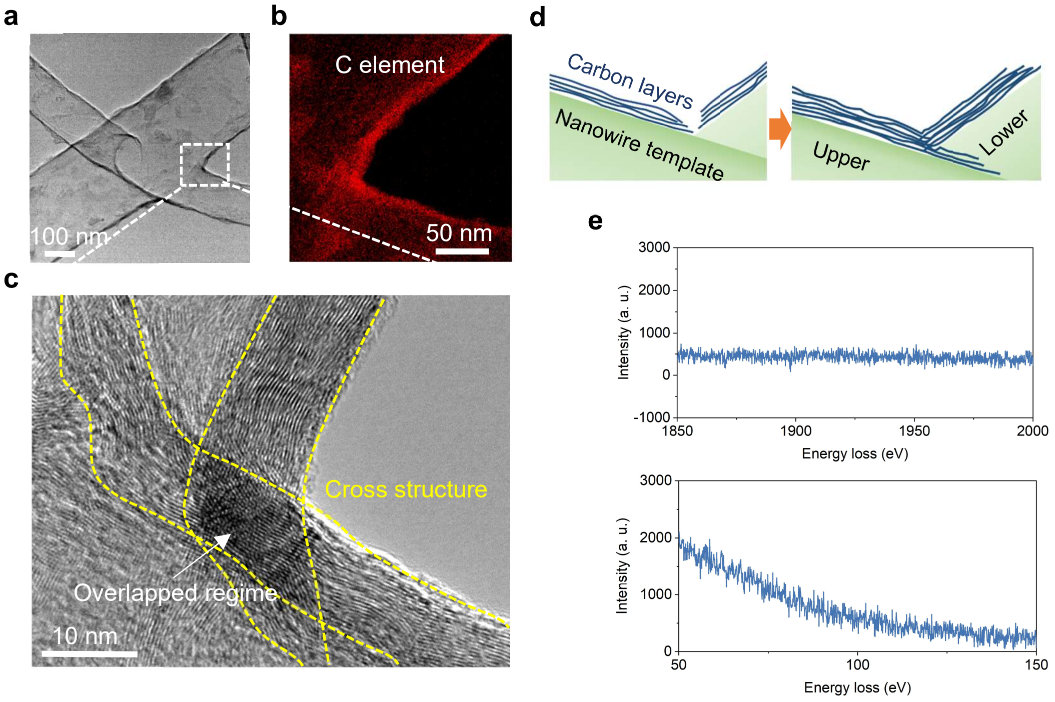

Supplementary Fig. 6. Microstructure of the joint between two carbon tubes. (a) TEM image of the joint. (b) Corresponding EDS mapping. (c) High-resolution TEM image. (d) Schematic diagram of the formation of overlapped regime in the joint of two adjacent carbon tubes. (e) EELS spectrum curves of a tube with energy loss ranging from 50–150 and 1,850–2,000 eV, respectively, suggesting no Si element in the tube (the characteristic peaks of Si element are at approximately 90 and 1,950 eV).

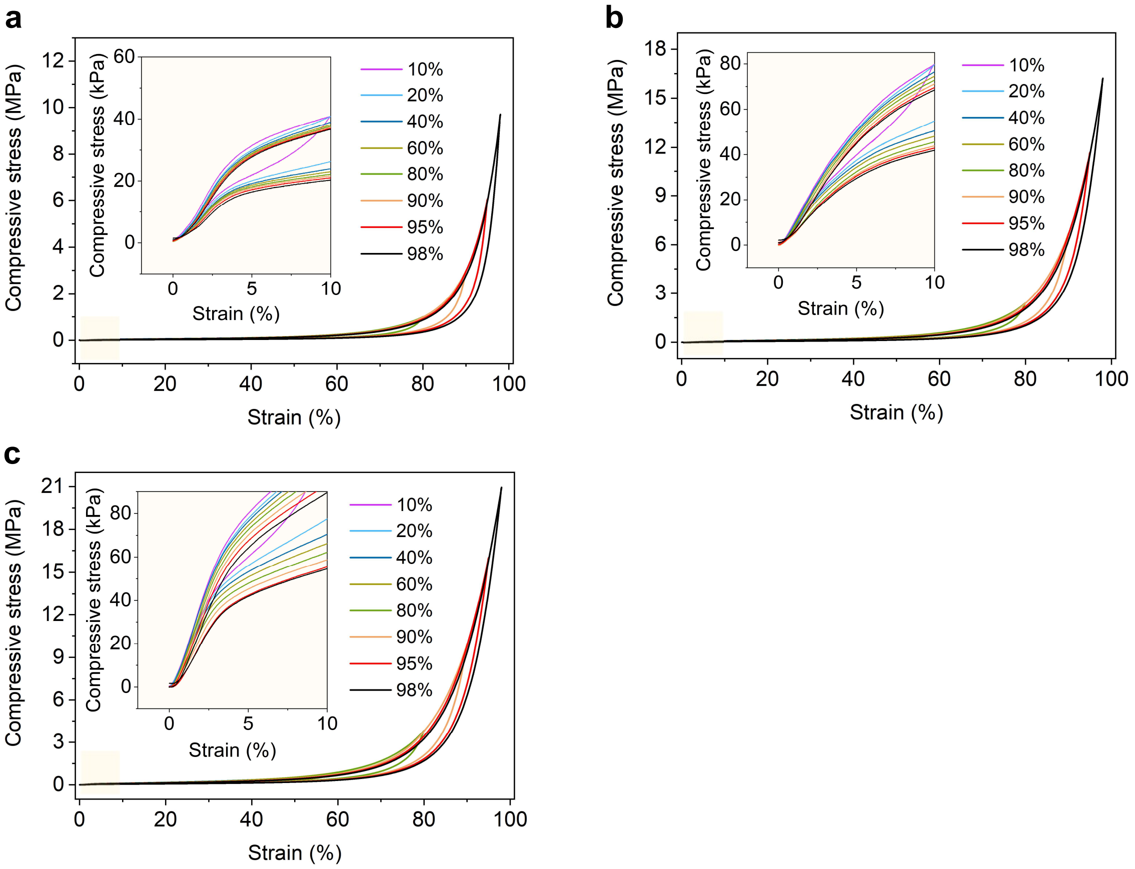

Supplementary Fig. 7. Compression stress vs. strain curves of CTAs with different density in a range of ~20–40 mg cm^{-3} using ~200 mg cm^{-3} SiC nanowire aerogels as template. (a) 19.7 mg cm^{-3}. (b) 31.5 mg cm^{-3}. (c) 43.2 mg cm^{-3}.

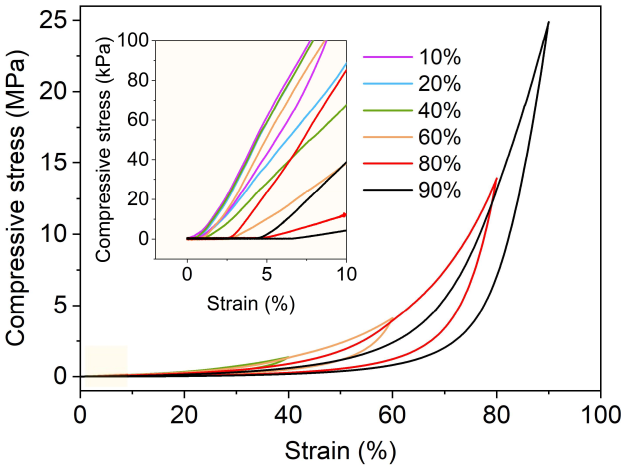

Supplementary Fig. 8. Compressive stress vs. strain curves of CTAs with a density of 22.4 mg cm^{-3} using ~300 mg cm^{-3} SiC nanowire aerogels as template. Approximately 5% permanent deformation is present when being compressed to ε = 90%.

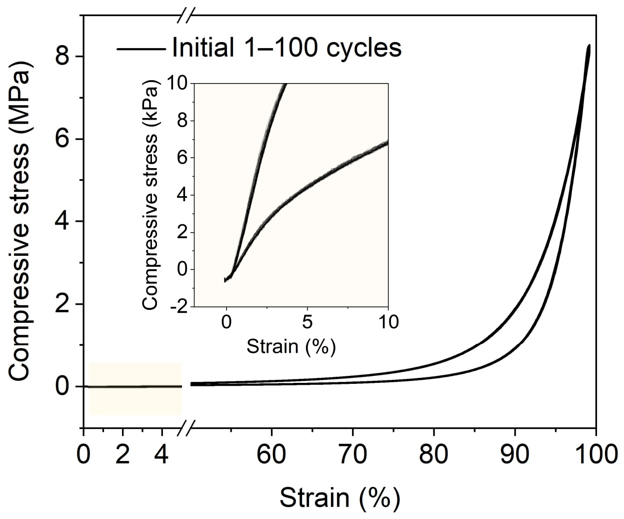

Supplementary Fig. 9. Compressive stress–strain curves of CTAs with density of 12.9 mg cm^{-3} at 99% strain for 100 cycles. After cycling, almost no compressive stress loss and permanent deformation are present.

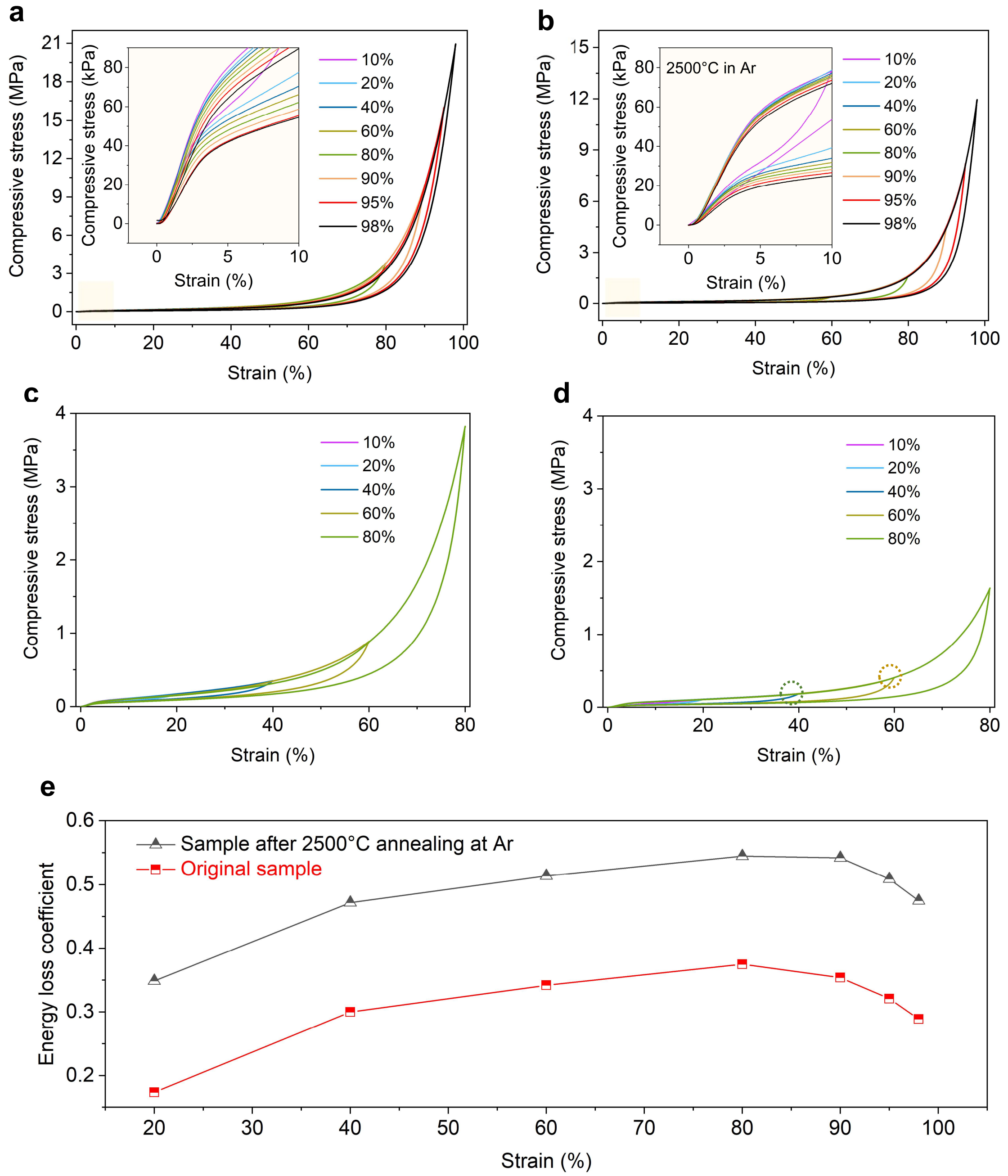

Supplementary Fig. 10. Compression behaviors of the CTA with a density of 43.2 mg cm^{-3} before and after 2500 °C annealing in Ar. (a) Stress vs. strain curves of the sample without annealing. (b) Stress vs. strain curves of the sample after annealing. (c) Enlarged image of (a) at a maximum strain of ε = 80%. (d) Enlarged image of (b) at a maximum strain of ε = 80%. (e) Energy loss coefficient of the sample before and after 2500 °C annealing.

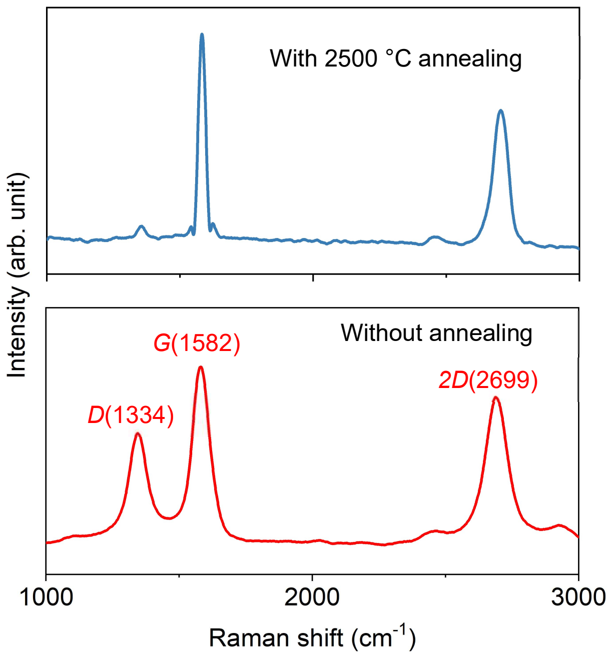

Supplementary Fig. 11. Raman spectrums of CTAs before and after 2500 °C annealing in Ar. Three typical peaks are observed, namely, *D* peak at 1334 cm^{-1}, *G* peak at 1582 cm^{-1}, and *2D* peak at 2699 cm^{-1}.

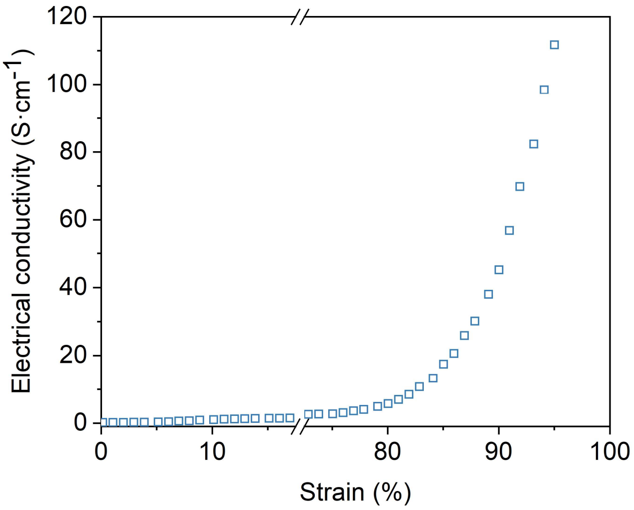

Supplementary Fig. 12. Electrical conductivity of a CTA with a density of 13.4 mg cm^{-3} when compressed to ε = 95%. The conductivity is tuned between 0.12 to 111.69 S cm^{-1}.

Supplementary Table 1. Compressive behaviors of CTAs compared to other elastic materials.

Material	Strain (%)	Stress (MPa)	Stress retention (%)	Cycle	Reference
CNT aerogels	60	0.19	100	2,000	*Nat. Nanotechnol.* (2012)[2]
CNT aerogels	30<br>90	0.025<br>0.125	70<br>92	1,000,000<br>10,000	*Chem*. (2019)[3]
GO foams	50	0.01	90	100	*Angew. Chem.* (2015)[4]
GO/$Ti_3C_2T_x$ foams	60	0.015	93	100	*ACS Nano*. (2021)[5]
GO foams	20<br>50<br>80	0.0005<br>0.003<br>0.025	97<br>86<br>60	1,000,000<br>250,000<br>10,000	*Nat. Commun*. (2016)[6]
Graphene aerogels	50<br>80	0.004<br>0.018	95<br>82	1,000<br>1,000	*Nat. Commun*. (2022)[7]
Graphene aerogels	50	0.004	70	5	*Adv. Mater.* (2013)[8]
Graphene aerogels	99	1	93	100	*Adv. Mater*. (2016)[9]
Graphene aerogels	50<br>50<br>90<br>90	0.005<br>0.005<br>0.14<br>0.12	92<br>45<br>77<br>36	100<br>100<br>100<br>100	*ACS Nano*. (2017)[10]
Graphene aerogels	80	0.1	81	1,000	*Nat. Commun*. (2015)[11]
Graphene aerogels	80	0.02	76	10	*Nat. Commun*. (2012)[12]
Graphene aerogels	50	0.015	90	1,000	*Carbon*. (2018)[13]
Graphene aerogels	90	0.01	92	100,000	*Sci. Adv.* (2020)[14]
Graphene aerogels	92	4500	N/A	1	*Commun. Phys*. (2022)[15]
Graphene aerogels	~99.8<br>~99	1000<br>40	N/A<br>~97	1<br>10	*Nanoscale*. (2018)[16]
Graphene aerogels	80	0.02	~90	10	*Carbon*. (2018)[17]
rGO aerogels	95	0.07	82	100	*Adv. Mater*. (2019)[18]
rGO aerogels	50<br>50	0.02<br>0.015	90<br>85	15<br>15	*ACS Nano*. (2018)[19]
rGO aerogels	50<br>80	0.00015<br>0.0011	85<br>84	10,000<br>100	*Adv. Funct. Mater*. (2022)[20]

rGO aerogels	99<br>90<br>70	0.016<br>0.0035<br>0.0025	50<br>42<br>55	10<br>100<br>10,000	*Adv. Mater.* (2018)[21]
Wood-derived carbon sponges	40	0.006	93	100	*ACS Nano*. (2018)[22]
Wood-derived carbon sponges	50	0.008	100	10,000	*Chem*. (2018)[23]
Wood-derived carbon sponges	50<br>90<br>95	0.0025<br>0.025<br>0.078	79<br>68<br>65	30,000<br>100<br>50	*Adv. Funct. Mater*. (2020)[24]
Carbon nanofiber foams	40<br>60<br>80	0.005<br>0.008<br>0.015	90<br>96<br>96	1,000,000<br>100,000<br>10,000	*Adv. Mater*. (2020)[25]
PAN nanofiber aerogels	20<br>60	0.004<br>0.012	100<br>75	100<br>1,000	*Nat. Commun*. (2014)[26]
BN nanosheet aerogels	90	0.045	86	100	*Science*. (2019)[27]
SiC nanowire aerogels	60	0.016	94	1,000	*ACS Nano*. (2018)[28]
ZrO_2/Al_2O_3 nanofiber aerogels	60	0.06	69	1,000	*ACS Nano*. (2020)[29]
SiC/Si_3N_4 nanowire aerogels	35	0.002	73	400	*Nano Lett*. (2021)[30]
$TiO_2/ZrO_2/BaTiO_3$ nanofiber aerogels	23	0.003	66	100	*Sci. Adv*. (2017)[31]
ZrO_2/zircon nanofiber aerogels	50	0.0016	93	1,000	*Nature*. (2022)[32]
CTA	99<br>99	8.2<br>8.2	100<br>98.7	100<br>1,000	This work

*The density of the listed CTA is 12.9 mg cm^{-3}.

Supplementary References

1. Bruley, J., Williams, D. B., Cuomo, J. J. & Pappas, D. P. Quantitative near-edge structure analysis of diamond-like carbon in the electron microscope using a two-window method. *J. Microsc*. **180**, 22-32 (1995).
2. Kyu, H. K., Youngseok, O. & Islam, M. F. Graphene coating makes carbon nanotube aerogels superelastic and resistant to fatigue. *Nat. Nanotechnol*. **7**, 562–566 (2012).
3. Zhan, H. J.*et al*. Biomimetic carbon tube aerogel enables super-elasticity and thermal insulation. *Chem*. **5**, 1871–1882 (2019).
4. Wang, X. *et al*. Scalable template synthesis of resorcinol-formaldehyde/graphene oxide composite aerogels with tunable densities and mechanical properties. *Angew. Chem*. 8, 2397–

2401 (2015).
5. Jiang, D. *et al*. Superelastic $Ti_3C_2T_x$ MXene-based hybrid aerogels for compression-resilient devices. *ACS Nano*. **15**, 5000–5010 (2021).
6. Gao, H. *et al*. Super-elastic and fatigue resistant carbon material with lamellar multi-arch microstructure. *Nat. Commun*. **7**, 1–8 (2016).
7. Wu, M.M. *et al*. Superelastic graphene aerogel-based metamaterials. *Nat. Commun*. **13**, 4561 (2022).
8. Hu, H., Zhao, Z., Wan, W., Gogotsi, Y. & Qiu, J. Ultralight and highly compressible graphene aerogels. *Adv. Mater*. **15**, 2219–2223 (2013).
9. Xu, X. *et al*. Naturally dried graphene aerogels with superelasticity and tunable Poisson's ratio. *Adv. Mater*. **28**, 9223–9230 (2016).
10. Yang, M. *et al*. Biomimetic architectured graphene aerogel with exceptional strength and resilience. *ACS Nano*. **11**, 6817–6824 (2017).
11. Wu, Y. *et al*. Three-dimensionally bonded spongy graphene material with super compressive elasticity and near-zero Poisson’s ratio. *Nat. Commun*. **6**, 1–9 (2015).
12. Qiu, L., Liu, J. Z., Chang, S. L., Wu, Y. & Li, D. Biomimetic superelastic graphene-based cellular monoliths. *Nat. Commun*. **3**, 1–7 (2012).
13. Liu, J. *et al*. Superelastic and multifunctional graphene-based aerogels by interfacial reinforcement with graphitized carbon at high temperatures. *Carbon*. **132**, 95–103 (2018).
14. Pang, K. *et al*. Hydroplastic foaming of graphene aerogels and artificially intelligent tactile sensors. *Sci. Adv*. **6**, eabd4045 (2020).
15. Šilhavík, M. *et al*. J. Anomalous elasticity and damping in covalently cross-linked graphene aerogels. *Commun. Phys*. **5**, 27 (2022).
16. Li, C., Ding, M., Zhang, B., Qiao, X. & Liu, C. Graphene aerogels that withstand extreme compressive stress and strain. *Nanoscale*. **10**, 18291–18299 (2018).
17. Li, X. H. *et al*. Vertically aligned, ultralight and highly compressive all-graphitized graphene aerogels for highly thermally conductive polymer composites. ***Carbon***. 140, 624-633 (2018).
18. Peng, M. *et al*. 3D printing of ultralight biomimetic hierarchical graphene materials with exceptional stiffness and resilience. *Adv. Mater*. **31**, 1902930 (2019).
19. Wang, C. *et al*. Freeze-casting produces a graphene oxide aerogel with a radial and centrosymmetric structure. *ACS Nano*. **12**, 5816–5825 (2018).
20. Liu, H. *et al*. Multifunctional superelastic, superhydrophilic, and ultralight nanocellulose-based composite carbon aerogels for compressive supercapacitor and strain sensor. *Adv. Funct. Mater*. **32**, 2113082 (2022).
21. Zhuo, H. *et al*. A supercompressible, elastic, and bendable carbon aerogel with ultrasensitive detection limits for compression strain, pressure, and bending angle. *Adv. Mater.* **30**, 1706705 (2018).
22. Guan, H., Cheng, Z. & Wang, X. Highly compressible wood sponges with a spring-like

lamellar structure as effective and reusable oil absorbents. *ACS Nano*. **12**, 10365–10373 (2018).

23. Chen, C. *et al*. Scalable and sustainable approach toward highly compressible, anisotropic, lamellar carbon sponge. *Chem*. **4**, 544–554 (2018).
24. Chen, Z. *et al*. Wood-derived lightweight and elastic carbon aerogel for pressure sensing and energy storage. *Adv. Funct. Mater*. **30**, 1910292 (2020).
25. Li, C. *et al*. Temperature-invariant superelastic and fatigue resistant carbon nanofiber aerogels. *Adv. Mater*. **2**, 1904331 (2020).
26. Si, Y. *et al*. Ultralight nanofibre-assembled cellular aerogels with superelasticity and multifunctionality. *Nat. Commun*. **5**, 1–9 (2014).
27. Xu, X. *et al*. Double-negative-index ceramic aerogels for thermal superinsulation. *Science*. **363**, 723–727 (2019).
28. Su, L. *et al*. Ultralight, recoverable, and high-temperature-resistant SiC nanowire aerogel. *ACS Nano*. **12**, 3103–3111 (2018).
29. Zhang, X. *et al*. Ultrastrong, superelastic, and lamellar multiarch structured ZrO_2-Al_2O_3 nanofibrous aerogels with high-temperature resistance over 1300 °C. *ACS Nano*. **14**, 15616–15625 (2020).
30. Zhang, X. *et al*. Three-dimensional reticulated, spongelike, resilient aerogels assembled by SiC/Si_3N_4 nanowires. *Nano Lett*. **21**, 4167–4175 (2021).
31. Wang, H. *et al*. Ultralight, scalable, and high-temperature-resilient ceramic nanofiber sponges. *Sci. Adv*. **3**, e1603170 (2017).
32. Guo, J. *et al*. Hypocrystalline ceramic aerogels for thermal insulation at extreme conditions. *Nature*. **606**, 909–916 (2022).
