# Supplementary material for: Highly cross-linked carbon tube aerogels with enhanced elasticity and fatigue resistance
Source: Nat Commun. 2023 Jun 1;14:3178. doi: 10.1038/s41467-023-38664-6 (PMC10235059; doi:10.1038/s41467-023-38664-6)
Supplement: Supplementary file 3 — Description of Additional Supplementary Information [file 41467_2023_38664_MOESM3_ESM.docx]

Additional Supplementary Files

**File Name: Supplementary Movie 1**

Description: Compression of a CTA up to 99% strain.

**File Name: Supplementary Movie 2**

Description: In situ TEM observation of carbon tube networks upon compression.

**File Name: Supplementary Movie 3**

Description: In situ TEM observation of a single carbon tube upon compression.

**File Name: Supplementary Movie 4**

Description: Breaking process of a *sp^3^* bond during bending of a tube.
